# Incidence and patterns of *ALK* FISH abnormalities seen in a large unselected series of lung carcinomas

**DOI:** 10.1186/1755-8166-5-44

**Published:** 2012-12-03

**Authors:** Zunyan Dai, JoAnn C Kelly, Aurelia Meloni-Ehrig, Marilyn L Slovak, Debra Boles, Nicole C Christacos, Christine R Bryke, Steven A Schonberg, Jennifer Otani-Rosa, Qiulu Pan, Albert K Ho, Heather R Sanders, Zhong J Zhang, Dan Jones, Philip N Mowrey

**Affiliations:** 1 Department of Cytogenetics, Quest Diagnostics Nichols Institute, 14225 Newbrook Drive, Chantilly, VA, 20151, USA; 2 Department of Cytogenetics, AmeriPath, 8150 Chancellor Dr. Suite 110, Orlando, FL, 32809, USA; 3 Department of Molecular Oncology, Quest Diagnostics Nichols Institute, 14225 Newbrook Drive, Chantilly, VA, 20151, USA; 4 Department of Pathology, Quest Diagnostics Nichols Institute, 14225 Newbrook Drive, Chantilly, VA, 20151, USA; 5 Department of Hematology and Oncology, Quest Diagnostics Nichols Institute, 33608 Ortega Highway, San Juan Capistrano, CA, 92675, USA

**Keywords:** *ALK* rearrangement, *ALK* amplification, FISH, *KRAS*, *EGFR*, Non-small cell lung cancer, Adenocarcinoma, Crizotinib

## Abstract

**Background:**

Anaplastic lymphoma receptor tyrosine kinase (*ALK*) gene rearrangements have been reported in 2-13% of patients with non-small cell lung cancer (NSCLC). Patients with *ALK* rearrangements do not respond to EGFR-specific tyrosine kinase inhibitors (TKIs); however, they do benefit from small molecule inhibitors targeting ALK.

**Results:**

In this study, fluorescence in situ hybridization (FISH) using a break-apart probe for the *ALK* gene was performed on formalin fixed paraffin-embedded tissue to determine the incidence of *ALK* rearrangements and hybridization patterns in a large unselected cohort of 1387 patients with a referred diagnosis of non-small cell lung cancer (1011 of these patients had a histologic diagnosis of adenocarcinoma). The abnormal FISH signal patterns varied from a single split signal to complex patterns. Among 49 abnormal samples (49/1387, 3.5%), 32 had 1 to 3 split signals. Fifteen samples had deletions of the green 5^′^ end of the *ALK* signal, and 1 of these 15 samples showed amplification of the orange 3^′^ end of the *ALK* signal. Two patients showed a deletion of the 3^′^*ALK* signal. Thirty eight of these 49 samples (38/1011, 3.7%) were among the 1011 patients with confirmed adenocarcinoma. Five of 8 patients with *ALK* rearrangements detected by FISH were confirmed to have *EML4-ALK* fusions by multiplex RT-PCR. Among the 45 *ALK*-rearranged samples tested, only 1 *EGFR* mutation (T790M) was detected. Two *KRAS* mutations were detected among 24 *ALK*-rearranged samples tested.

**Conclusions:**

In a large unselected series, the frequency of *ALK* gene rearrangement detected by FISH was approximately 3.5% of lung carcinoma, and 3.7% of patients with lung adenocarcinoma, with variant signal patterns frequently detected. Rare cases with coexisting *KRAS* and *EGFR* mutations were seen.

## Background

Lung cancer is the leading cause of cancer-related deaths in the US with an estimated 160,000 deaths attributed to lung cancer in 2012 [1]. Histologically, the World Health Organization (WHO) classifies lung cancer into non-small cell lung cancer (NSCLC, 85%) and small-cell lung cancer (15%). NSCLC is further divided into three major subgroups: adenocarcinoma, squamous cell carcinoma, and a less well-characterized group of large cell carcinoma (40%, 30% and 15% of all lung cancer cases in the United States, respectively). Based on the patient’s medical status and stage of disease, there are four general treatment strategies, including surgery, radiation therapy, chemotherapy, and/or targeted therapy. The use of targeted kinase inhibitors is based on the presence of specific genetic alterations, such as activating mutations in the epidermal growth factor receptor *(EGFR*) or activating chromosomal fusions involving the *ALK* kinase, and can be highly effective if the tumors harbor these associated molecular alterations [2]. Therefore, molecular tests are routinely performed to identify mutations in oncogenes in lung cancer, including *EGFR* and *ALK*; to identify those patients with a high likelihood of response to targeted therapy; and reduce unnecessary side effects of ineffective treatments. The mutation status of *KRAS* is usually also assessed since tumors that show such mutations do not respond to targeted kinase therapies.

*ALK* gene rearrangements have been reported in 2% to 13% of patients with NSCLC [2]. Most patients with *ALK* rearrangements typically have adenocarcinomas and are younger patients with minimal to no smoking history. *ALK*-rearranged tumors typically lack *EGFR* and *KRAS* mutations. Recent studies have demonstrated that lung cancers harboring *ALK* rearrangements do not respond to EGFR-specific tyrosine kinase inhibitors, but benefit from a small molecule inhibitor targeting the ALK kinase (ie, crizotinib [Xalkori®, Pfizer, Inc.]).

**Figure 1:**
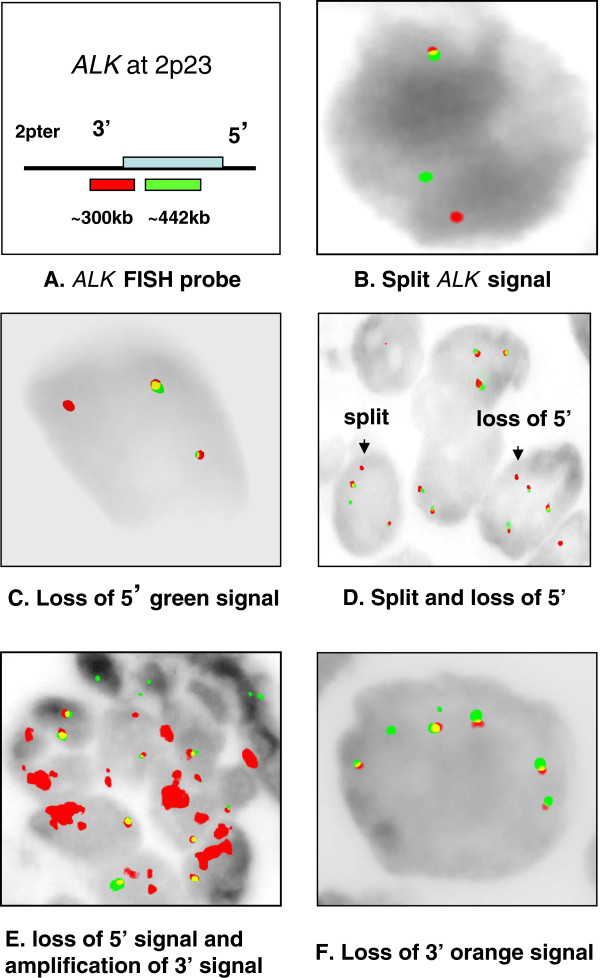
**FISH analysis with the *ALK *dual-color break-apart probe shows variable rearrangement patterns.A**) The FDA approved break-apart FISH probe (Abbott Molecular) at 2p23 to detect *ALK* rearrangements. The 5^′^ end of the *ALK* gene is labeled with SpectrumGreen and the 3^′^ end of the *ALK* gene is labeled with SpectrumOrange. **B**) Samples had 1 to 3 split signals. **C**) Deletion of the green 5^′^ end of the *ALK* signal. **D**) Coexistence of polysomy, split signal, and deletion of the 5^′^ end of the *ALK* signal. **E**) Malignant cells in one patient with adenocarcinoma showing deletion of the 5^′^ end of the *ALK* signal and amplification of the 3^′^ end signals. **F**) Two patients showed a deletion of the orange 3^′^ signal and considered negative for an *ALK* rearrangement.

Fluorescence in situ hybridization (FISH) with a multicolor break-apart *ALK* probe is the currently FDA-approved method for screening for *ALK* gene rearrangements (Figure 1A). Exon-scanning reverse-transcription PCR methods have also been developed. Inversion of the short arm of chromosome 2 is the most common rearrangement associated with the *ALK* gene in lung cancer. This rearrangement leads to fusion between the 5^′^ end of the echinoderm microtubule-associated protein-like 4 (*EML4)* gene (located at 2p21) and the 3^′^ end of the *ALK* gene (at 2p23): the *EML4-ALK* fusion. Alternate *ALK* FISH signal patterns which may indicate other activating rearrangements also occur and are studied here.

The reasons for the variability in the detection frequency of *ALK* gene rearrangements in NSCLC could be due to a variety of technical and biologic influences. These include biases due to small sample size, variations in the performance and interpretation of *ALK* FISH studies, and the relative proportions of smoking-related cases which usually lack both *EGFR* and *ALK* mutations. In a prior large screening study, *ALK* rearrangement was detected in 5.5% of 1500 cases of NSCLC [3]. In this study, we examined the frequency and patterns of *ALK* rearrangements in clinical specimens from a large cohort of lung cancer patients from both primary and tertiary settings across the United States referred for molecular profiling.

## Results

*ALK* FISH was performed on an unselected series that included 1387 samples from patients with NSCLC. Overall, 49 of the 1387 tumor samples (3.5%) had altered *ALK* signals. Thirty eight of these 49 samples (38/1011, 3.7%) were from patients with confirmed adenocarcinomas (Table 1). In 32 cases, there was the expected pattern of one split orange and green signal indicative of an *ALK* gene rearrangement and one single fusion signal from the intact *ALK* gene (Figure 1B). Among variant signals, the most commonly seen (15 cases) was deletion of the 5^′^*ALK* (green signal, Figure 1C). More complex FISH patterns included normal split patterns along with loss of the 5^′^ signal; this was often seen in tumors that showed gains of intact *ALK* signals consistent with polysomy (Figure 1D).

**Table 1:** **Frequency of *ALK *rearrangements in lung cancer patients**

	**All patients**	**Adenocarcinoma as submitting diagnosis**
*ALK* rearrangement	49^a^	38^b^
Total cases	1387	1011
% *ALK* rearrangement	3.5%	3.7%

^a^ One patient had large cell carcinoma and 10 patients were diagnosed as carcinomas without further typing. Two of the 49 patients had a deletion of 3^′^ end signal, which should be regarded as negative for *ALK* rearrangement. ^b^One patient showed a deletion of 5^′^*ALK* and amplification of the 3^′^ end signal.

One of the 15 patients with deletion of the 5^′^*ALK*, also showed 5-25 copies of the 3^′^*ALK* (high level amplification, Figure 1E). Two samples showed a deletion of the 3^′^*ALK* (orange signal, Figure 1F). According to the new FDA-approved Vysis FISH *ALK* break-apart probe kit guidelines, patients with deletion of the 3^′^*ALK* split signal should be considered negative for rearrangements, because the tyrosine kinase domain of *ALK* is located in the 3^′^ region of the *ALK* gene.

**Figure 2:**
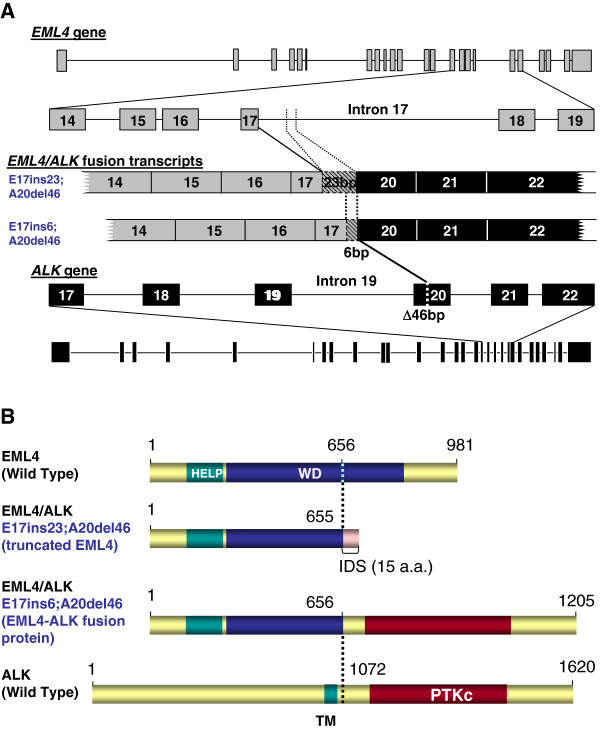
**Characterization of novel *EML4-ALK *transcription variants in a patient with lung adenocarcinoma.** This patient had a typical positive break-apart FISH signal pattern of the *ALK* gene. **A)** Two transcription variants were detected, derived from a fusion of *EML4* exon 17 to the 3^′^ end portion of *ALK* exon 20, with 46 base pairs deleted from the 5^′^ end of *ALK* exon 20. These two fusion transcripts were from the same gene rearrangement with either a 23-nucleotide or a 6-nucleotide insertion from *EML4* intron 17. **B)** Putative proteins from *EML4-ALK* fusion transcripts. The transcript with an insertion of 23 nucleotides produced a truncated EML4 protein and no EML4-ALK fusion protein, as a result of an early stop codon. However, the transcript with an insertion of 6 nucleotides produced an EML4-ALK fusion protein, containing the N-terminal region of EML4 and the C-terminal region of ALK with the protein tyrosine kinase domain (PTKc).

To investigate the significance of variant signal patterns, we also performed exon scanning multiplex RT-PCR to identify *EML4-ALK* fusion transcripts in a subset of both typical and variant cases. Five of these samples (3 with break apart and 2 with loss of 5^′^*ALK*) were confirmed positive for *EML4-ALK* fusion. Among these, one was positive for fusion variant 1, one for variant 3a, and two for both variants 3a and 3b. These are the most common *ALK* fusion variants reported by other investigators [2]. In addition, new transcription variants were identified, derived from a fusion of *EML4* exon 17 to the 3^′^ end portion of *ALK* exon 20 with two different small insertions (Figure 2 for gene structures and putative proteins, and Additional file 1 for nucleotide sequences and amino acid sequences). The 3 samples that did not show *EML4-ALK* fusion by PCR had FISH patterns with deletion of the 5^′^*ALK* probe.

Among the 45 *ALK*-rearranged samples tested for mutations in exons 18-21 of the *EGFR* gene, only one mutation, T790M, was detected in a patient with a typical positive break-apart FISH signal pattern of the *ALK* gene. Two *KRAS* mutations were detected among 24 *ALK*-positive samples examined, G12F in a patient with a deletion of the green 5^′^ end of the *ALK* signal and G13C in the sample that showed amplification of the 3^′^*ALK* region (Figure 1E).

## Discussion

In this study, the frequency of *ALK* gene rearrangements detected using a dual-color break-apart FISH probe was 3.7% of adenocarcinoma cases. This is somewhat lower than earlier studies that generally had smaller number of patients, but close to 3.9% which was observed in two cohorts of 720 and 1121 lung adenocarcinomas from two series in Japan [4,5]. Our results are consistent with previous studies where *ALK* rearrangements have been largely (but not completely) restricted to adenocarcinomas that lack *EGFR* or *KRAS* mutations.

Here, we show that the *ALK* FISH signal patterns may vary from a single split signal to very complex signal patterns. Gains of intact *ALK* signals as well as typical and variant hybridization patterns for *ALK* gene rearrangements were observed simultaneously in some samples likely due to intratumoral heterogeneity. Although polysomy of chromosome 2 did occur frequently in NSCLC (and was not regarded as evidence of an *ALK*-specific rearrangement), high-level amplification of the *ALK* fusion signal was a rare event.

The current study and a previous report using the same PCR method [6] showed correlation between PCR and FISH for detection of *EML4-ALK* fusions even when loss of 5^′^ green signals was seen. In our study, 5 samples (3 with break apart and 2 with loss of 5^′^*ALK*) were positive for an *EML4-ALK* fusion by multiplex RT-PCR. This is similar to a previous study that an *EML4-ALK* fusion was detected by RT-PCR in 22 out of 31 patients with FISH-positive *ALK* rearrangements [3]. However, FISH also detected variant *ALK* signal patterns in three cases that were negative for *EML4-ALK* fusion by PCR; this may be indicative of translocations involving *ALK* and genes other than *EML4*[3], or other mechanisms of *ALK* activation. The general correlation between the two methods supports using loss of the 5^′^*ALK* signal as presumptive evidence of an *ALK* gene rearrangement.

*EML4-ALK* and other 2p23/*ALK* gene rearrangements lead to a constitutively activated ALK kinase, which confers sensitivity of NSCLC tumors to ALK inhibitors [3]. Lung cancer patients with *ALK* rearrangements have been shown to have significant reductions in tumor burden in response to treatment with the ALK inhibitor crizotinib, which led to its accelerated FDA approval in the United States in 2011 [3,7,8]. Crizotinib has also been shown to have activity against the 1-2% of patients with NSCLC that show rearrangements of another receptor tyrosine kinase gene, *ROS1*, located at 6q22 [5,9].

Patients who harbor *ALK* rearrangements do not benefit from treatment with the EGFR-specific tyrosine kinase inhibitors showing that the EGFR inhibition is bypassed. The clinical behavior of NSCLC cases with variant *ALK* signal patterns is less clear. Amplification of the *ALK* fusion has been seen in a patient undergoing crizotinib therapy [10] and may be a sign of developing resistance. But the response of untreated NSCLC with *ALK* gene amplification is not yet clear. Given the large number of signaling pathways that are influenced by ALK fusion products [7], the behavior and appropriate treatment of NSCLC cases that have variant *ALK* signal patterns also require further study.

## Conclusions

Use of a break-apart *ALK* FISH probe is an effective method for assessing *ALK* gene rearrangement status in routinely submitted formalin fixed paraffin-embedded NSCLC tumor samples from a wide variety of tissue sources and clinical settings. Variant *ALK* FISH signals usually, but not always, represent *EML4* –*ALK* fusions.

## Methods

### Patient samples

Samples from 1387 lung cancer patients (1011 with a submitting diagnosis of adenocarcinoma) were included in this study. Most were small transbronchial biopsies, or cell blocks of lymph node aspirates or malignant pleural or pericardial effusions. These samples were consecutively submitted for *ALK* rearrangement testing. The presence of lung cancer cells was verified by pathological examination of a hematoxylin-eosin-stained section adjacent to the slide used for FISH analysis. Mutation studies for *EGFR* and *KRAS* were also performed if sufficient tumor was available, as was *EML4-ALK* RT-PCR.

### *ALK* FISH

FISH using a dual-color break-apart probe for *ALK* was performed on paraffin-embedded tissue sections according to the Vysis *ALK* Break Apart FISH Probe Kit protocol (Abbott Molecular, Des Plaines, IL), using the BX51/BX52 Olympus fluorescence microscope (Olympus, Richardson, TX). The SenSys® CCD camera (Photometrics, Tucson, AZ) was used to capture selected images. The occurrence of an *ALK* rearrangement (*ALK* positive) was concluded if >15% of tumor cells showed split orange and green signals and/or deletion of green signals; otherwise, the specimen was classified as negative for *ALK* rearrangement.

### *EGFR* and *KRAS* mutations

Sanger sequencing of dissected lung cancer samples was performed to detect mutations in *EGFR* and *KRAS*. DNA was PCR amplified for exons 18-21 of *EGFR* and exons 1 and 2 of *KRAS*. The PCR products were purified and sequenced on a DNA sequencer (ABI; Carlsbad, CA).

### Exon scanning RT-PCR for *EML4-ALK* fusion transcripts

RNA was extracted from formalin fixed paraffin-embedded sections, reverse transcribed and PCR amplified using one-step RT-PCR kit and products detected using fragment analysis on ABI 3730 genetic analyzer, as previously described [6]. The assay utilized 22 *EML4* exon primers and a single *ALK* reverse primer to detect a variety of inversion variants. Fusion types were identified based on expected sizes of the PCR products, with the new variant identified by standard dideoxy chain-termination DNA sequencing of the abnormally-sized cDNA PCR product, as described [6].

## Abbreviations

ALK: Anaplastic lymphoma receptor tyrosine kinase; EML4: Echinoderm microtubule-associated protein-like 4; EGFR: Epidermal growth factor receptor; KRAS: Kirsten rat sarcoma viral oncogene homolog; NSCLC: Non-small cell lung cancer; TKIs: Tyrosine kinase inhibitors; FISH: Fluorescence in situ hybridization; ROS1: c-ros oncogene 1, receptor tyrosine kinase; RT-PCR: Reverse transcription polymerase chain reaction.

## Competing interests

The authors declare that they have no competing interests.

## Authors’ contributions

ZD, JCK, AME, MLS, DB, NCC, CRB, SAS and PNM participated in the *ALK* FISH analysis, ZD, QP, AKH and DMJ interpreted the mutation results of *EGFR* and *KRAS*. HSR and ZJZ performed the RT-PCR test. JOR participated in the coordination and helped to draft the manuscript. ZD wrote the manuscript with input from co-authors. All authors read and approved the final manuscript.

## Supplementary Material

Additional file 1 **Dai et. al., Supplemental data for nucleotide sequences and putative amino acid sequences of new *EML4-ALK *transcription variants in a patient with lung adenocarcinoma.** Click here for file
