# Supplementary material for: Incidence and patterns of ALK FISH abnormalities seen in a large unselected series of lung carcinomas
Source: Mol Cytogenet. 2012 Dec 3;5:44. doi: 10.1186/1755-8166-5-44 (PMC3576271; doi:10.1186/1755-8166-5-44)
Supplement: Additional file 1 — Dai et. al., Supplemental data for nucleotide sequences and putative amino acid sequences of new EML4-ALK transcription variants in a patient with lung adenocarcinoma. [file 1755-8166-5-44-S1.doc]

**Dai et. al., Supplemental data for nucleotide sequences and putative amino acid sequences of new *EML4-ALK* transcription variants in a patient with lung adenocarcinoma.** The patient had a typical positive break-apart FISH signal pattern of the *ALK* gene. Two transcription variants were detected, derived from a fusion of *EML4* exon 17 to the 3′ end portion of *ALK* exon 20, with 46 base pairs deleted from 5′ end of *ALK* exon 20. These two fusion transcripts were from a same gene rearrangement with a 23-nucleotide or 6-nucleotide insertion from *EML4* intron 17. Nucleotide sequences and putative amino acid sequences of these fusion variants are listed below.

Nucleotide sequences of *EML4-ALK* fusion transcripts from *EML4* exon 17 and 3′ end portion of *ALK* exon 20. The fusion site sequence of the transcript with an insertion of 23 nucleotides is shown below (E17ins23;A20del46, Black, EML4 exon 17; red, EML4 partial intron 17; blue, ALK exon 20):

…GAACCAGGACACTGTGCAGATTTTCATCCAAGTGGCACAGTGGTGGCCATAGGAACGCACTCAGGCAGcatactatgtatacaagggagttGCAGAGCCCTGAGTACAAGCTGAGCAAGCTCCGCACCTCGACCATCATGACCGACTACAACCCCAACTACTGCTTTGCTGGCAAGACCTCCTCCATCAGTGACCTGAAGGAGGTGCCGCGGAAAAAC…

The fusion site sequence of the transcript with an insertion of 6 nucleotides (E17ins6;A20del46, Black, EML4 exon 17; red, EML4 partial intron 17; blue, ALK exon 20):

…GAACCAGGACACTGTGCAGATTTTCATCCAAGTGGCACAGTGGTGGCCATAGGAACGCACTCAGGCAGggagttGCAGAGCCCTGAGTACAAGCTGAGCAAGCTCCGCACCTCGACCATCATGACCGACTACAACCCCAACTACTGCTTTGCTGGCAAGACCTCCTCCATCAGTGACCTGAAGGAGGTGCCGCGGAAAAAC…

Putative amino acid sequences from *EML4-ALK* fusion transcripts. The amino acid sequence from the transcript with a 23-nucleotide insertion produced a truncated EML4 protein and no EML4-ALK fusion protein, as a result of an early stop codon (black font, EML4; red font, nonsense): MDGFAGSLDDSISAASTSDVQDRLSALESRVQQQEDEITVLKAALADVLRRLAISEDHVASVKKSVSSKGQPSPRAVIPMSCITNGSGANRKPSHTSAVSIAGKETLSSAAKSGTEKKKEKPQGQREKKEESHSNDQSPQIRASPSPQPSSQPLQIHRQTPESKNATPTKSIKRPSPAEKSHNSWENSDDSRNKLSKIPSTPKLIPKVTKTADKHKDVIINQEGEYIKMFMRGRPITMFIPSDVDNYDDIRTELPPEKLKLEWAYGYRGKDCRANVYLLPTGEIVYFIASVVVLFNYEERTQRHYLGHTDCVKCLAIHPDKIRIATGQIAGVDKDGRPLQPHVRVWDSVTLSTLQIIGLGTFERGVGCLDFSKADSGVHLCVIDDSNEHMLTVWDWQKKAKGAEIKTTNEVVLAVEFHPTDANTIITCGKSHIFFWTWSGNSLTRKQGIFGKYEKPKFVQCLAFLGNGDVLTGDSGGVMLIWSKTTVEPTPGKGPKGVYQISKQIKAHDGSVFTLCQMRNGMLLTGGGKDRKIILWDHDLNPEREIEVPDQYGTIRAVAEGKADQFLVGTSRNFILRGTFNDGFQIEVQGHTDELWGLATHPFKDLLLTCAQDRQVCLWNSMEHRLEWTRLVDEPGHCADFHPSGTVVAIGTHSGSILCIQGSCRALSTS

The amino acid sequence of the EML4-ALK fusion protein from the transcript with a 6-nucleotide insertion (E17ins6;A20del46, black font, EML4; red font, insertion from EML4 intron 17; blue font, ALK):

MDGFAGSLDDSISAASTSDVQDRLSALESRVQQQEDEITVLKAALADVLRRLAISEDHVASVKKSVSSKGQPSPRAVIPMSCITNGSGANRKPSHTSAVSIAGKETLSSAAKSGTEKKKEKPQGQREKKEESHSNDQSPQIRASPSPQPSSQPLQIHRQTPESKNATPTKSIKRPSPAEKSHNSWENSDDSRNKLSKIPSTPKLIPKVTKTADKHKDVIINQEGEYIKMFMRGRPITMFIPSDVDNYDDIRTELPPEKLKLEWAYGYRGKDCRANVYLLPTGEIVYFIASVVVLFNYEERTQRHYLGHTDCVKCLAIHPDKIRIATGQIAGVDKDGRPLQPHVRVWDSVTLSTLQIIGLGTFERGVGCLDFSKADSGVHLCVIDDSNEHMLTVWDWQKKAKGAEIKTTNEVVLAVEFHPTDANTIITCGKSHIFFWTWSGNSLTRKQGIFGKYEKPKFVQCLAFLGNGDVLTGDSGGVMLIWSKTTVEPTPGKGPKGVYQISKQIKAHDGSVFTLCQMRNGMLLTGGGKDRKIILWDHDLNPEREIEVPDQYGTIRAVAEGKADQFLVGTSRNFILRGTFNDGFQIEVQGHTDELWGLATHPFKDLLLTCAQDRQVCLWNSMEHRLEWTRLVDEPGHCADFHPSGTVVAIGTHSGRELQSPEYKLSKLRTSTIMTDYNPNYCFAGKTSSISDLKEVPRKNITLIRGLGHGAFGEVYEGQVSGMPNDPSPLQVAVKTLPEVCSEQDELDFLMEALIISKFNHQNIVRCIGVSLQSLPRFILLELMAGGDLKSFLRETRPRPSQPSSLAMLDLLHVARDIACGCQYLEENHFIHRDIAARNCLLTCPGPGRVAKIGDFGMARDIYRASYYRKGGCAMLPVKWMPPEAFMEGIFTSKTDTWSFGVLLWEIFSLGYMPYPSKSNQEVLEFVTSGGRMDPPKNCPGPVYRIMTQCWQHQPEDRPNFAIILERIEYCTQDPDVINTALPIEYGPLVEEEEKVPVRPKDPEGVPPLLVSQQAKREEERSPAAPPPLPTTSSGKAAKKPTAAEVSVRVPRGPAVEGGHVNMAFSQSNPPSELHRVHGSRNKPTSLWNPTYGSWFTEKPTKKNNPIAKKEPHERGNLGLEGSCTVPPNVATGRLPGASLLLEPSSLTANMKEVPLFRLRHFPCGNVNYGYQQQGLPLEAATAPGAGHYEDTILKSKNSMNQPGP
